# *SMN1* c.5C>G (p.Ala2Gly) missense variant, a challenging molecular SMA diagnosis associated with mild disease, preserves SMN nuclear gems in patient-specific fibroblasts

**DOI:** 10.3389/fgene.2024.1406819

**Published:** 2024-07-30

**Authors:** Sara L. Cook, Christian Stout, Lindsey Kirkeby, Noemi Vidal-Folch, Devin Oglesbee, Linda Hasadsri, Duygu Selcen, Margherita Milone, Daniel Anderson, Nathan P. Staff

**Affiliations:** ^1^ Department of Laboratory Medicine and Pathology, Mayo Clinic, Rochester, MN, United States; ^2^ Center for Regenerative Medicine, Mayo Clinic, Rochester, MN, United States; ^3^ Department of Neurology, Mayo Clinic, Rochester, MN, United States; ^4^ Department of Neurology, Mayo Clinic Health System, La Crosse, WI, United States

**Keywords:** spinal muscular atrophy, exon 1, *SMN1*, c.5C>G, p.Ala2Gly, nuclear gems, diagnostic testing

## Abstract

**Introduction:**

Spinal muscular atrophy (SMA) is caused by homozygous loss of the *SMN1* gene with *SMN2* gene copy number correlating with disease severity. Rarely SMA is caused by a deletion on one allele and a pathogenic variant on the other. The pathogenic missense variant c.5C>G (p.Ala2Gly) correlates with a mild disease phenotype that does not correlate with *SMN2* copy number. In a mouse model the c.5C>G transgene produces SMN that is thought to form partially functional SMN complexes, but levels in humans have not yet been investigated.

**Methods:**

We identified two patients with mild SMA caused by a heterozygous deletion of *SMN1* and the heterozygous variant, c.5C>G. Molecular findings were confirmed with deletion/duplication analysis and Sanger sequencing. Skin fibroblasts were collected and cultured, and SMN expression was analyzed using immunofluorescence.

**Results:**

Two patients with slowly progressing mild weakness were confirmed to have heterozygous pathogenic missense variant c.5C>G and a heterozygous deletion of *SMN1*. Their clinical presentation revealed much milder disease progression than patients with matched *SMN2* copy number. Analysis of the patients’ fibroblasts revealed much higher numbers of SMN nuclear complexes than a patient with a homozygous *SMN1* deletion and matched *SMN2* copy number.

**Conclusions:**

These case reports reinforce that the rare c.5C>G variant causes mild disease. Furthermore, the analysis of SMA nuclear gems in patient samples supports the theory that the p.Ala2Gly SMN can form partially functional SMN complexes that may carry out essential cellular functions and result in mild disease.

## Introduction

Spinal muscular atrophy (SMA) is an autosomal recessive neurodegenerative disease which occurs in one out of every 10,000 live births (Pearn, 1978; Sugarman et al., 2012). SMA is characterized by degeneration of the alpha motor neurons of the spinal cord leading to progressive muscle weakness, and in cases of severe SMA, progression to respiratory failure and death before the age of two if disease-modifying therapies are not initiated (Burghes and Beattie, 2009; Faravelli et al., 2015). However, SMA is a clinically heterogeneous disease, historically divided into 4 different types, with Type I being the most severe of the four types. Patients with Type II SMA present with marked delay in gross motor development by 6–18 months of life, never become ambulatory, and suffer from progressive weakness, scoliosis, and restrictive lung disease (Faravelli et al., 2015). Patients with Type III SMA are ambulatory initially, but often require wheelchair assistance by adolescence (Faravelli et al., 2015). The mildest type of SMA, Type IV, generally presents with mild proximal limb girdle weakness that progresses slowly (Faravelli et al., 2015). Rarely patients exhibit disease more severe than Type I SMA and are considered to have Type 0 SMA. Type 0 SMA is characterized by diminished fetal movements detected prenatally and subsequent presentation at birth with asphyxia and severe hypotonia (MacLeod et al., 1999).

Most commonly SMA, with its four types, is caused by pathogenic variants in the survival motor neuron 1 (*SMN1*) gene. Humans also have the survival motor neuron 2 (*SMN2*) gene, which differs from *SMN1* gene by five nucleotides (Lefebvre et al., 1995; Burglen et al., 1996), all of which are in non-coding regions, except for the cysteine to thymine variation in exon 7 (Lefebvre et al., 1995; Burglen et al., 1996). Ultimately, this variation is translationally silent and the amino acid sequence of *SMN2* is identical to *SMN1*; however, the variation affects the alternative splicing such that 90% of transcripts are missing exon 7 and results in an unstable protein that is rapidly degraded (Cartegni and Krainer, 2002; Vitte et al., 2007; Burnett et al., 2009; Cho and Dreyfuss, 2010; Singh and Singh, 2018). Accordingly, there is an inverse relationship between *SMN2* copy number and disease severity. In general, Type 0 SMA have one copy of *SMN2*, Type I SMA patients have two copies of *SMN2*, Type II SMA patients have three copies of *SMN2*, Type III SMA patients have three or four copies of *SMN2*, and Type IV SMA patients have four or greater copies of *SMN2* (Feldkotter et al., 2002; Wadman et al., 2020). A small percent of SMA patients (non-5q SMA patients) have pathogenic variants in other genes, such as *BICD2*, *VRK1*, and *VAPB*, among others (Peeters et al., 2014).

Approximately 95% of affected SMA patients, regardless of type, have a homozygous deletion of *SMN1* exon 7 (Hahnen et al., 1995). Therefore, when a diagnosis of SMA is suspected, first-tier molecular analysis consists of deletion/duplication testing which utilizes techniques to quantify *SMN1* exon 7. Such techniques include multiplex ligation-dependent probe amplification (MLPA), quantitative PCR (qPCR), or droplet digital PCR (ddPCR). Often, these testing strategies will simultaneously quantify *SMN2* copy number. Five percent of SMA patients are compound heterozygotes and have a deletion on one allele and a more subtle pathogenic variant in the other allele (Wirth, 2000). Following this, if the results from MLPA, qPCR, or ddPCR methods return as negative in a patient with clinical and electrodiagnostic findings consistent with SMA, it is recommended to perform full gene analysis of *SMN1*. One percent of SMA patients have a loss of function *SMN1* missense variant (McAndrew et al., 1997; Wirth, 2000). Of these loss of function missense variants, some are reported to cause disease more severe or milder than predicted by *SMN2* copy number. The pathogenic missense variant c.5C>G (p.Ala2Gly) in compound heterozygosity with an *SMN1* deletion is known to correlate with a mild disease phenotype that does not correlate with *SMN2* copy number. These patients are indeed reported to be ambulatory into adolescence and adulthood even with low copy numbers of *SMN2* (Parsons et al., 1998; Bai et al., 2014; Yamamoto et al., 2014; Kubo et al., 2015; Mendonca et al., 2020).

The missense variant c.5C>G occurs in exon 1 of *SMN1*. Exon 1 of *SMN1* is identical to exon 1 of *SMN2*, making design of full gene analysis challenging. There are reports of efficient long-range PCR that can amplify exons 1-8 of *SMN1* (Kubo et al., 2015; Miller et al., 2023). This method utilizes the variation in exon 8 for primer design to specifically amplify *SMN1*, not *SMN2*. However, most clinical tests utilizing long range PCR only amplify exons 2a-7. This strategy utilizes the variation in intron 7 for primer design to specifically amplify *SMN1*, not *SMN2*. *SMN1* exon 1 is separately PCR-amplified. However, PCR primers will amplify both *SMN1* exon 1 and *SMN2* exon 1. Therefore, if a pathologic variant is detected in exon 1, a definitive molecular diagnosis cannot be made. In these cases, a clinician must use these molecular findings to supplement a clinical diagnosis of SMA.

Exon 1 of *SMN1* codes for a region just upstream of the Gemin-2 binding domain which is important for SMN complex assembly (Lorson and Androphy, 1998; Otter et al., 2007; Ogawa et al., 2009; Zhang et al., 2011). The SMN complex, which includes SMN, Gemin 2–8, and Unrip, plays an important role in small nuclear ribonucleoprotein (snRNP) assembly (Paushkin et al., 2002; Gubitz et al., 2004; Kolb et al., 2007). SMN complexes are thought to be involved in recycling and regeneration of spliceosomal U snRNPs (Pellizzoni et al., 1998). SMN complexes are also thought to be involved in pre-mRNA splicing by regulation of the Sm core protein composition of U snRNPs (Meister et al., 2001). This complex is observed in prominent nuclear dot-like structures termed gemini of coiled bodies, or “gems” (Liu and Dreyfuss, 1996).

Tissues collected from SMA patients, including lymphocytes, fibroblasts, and induced pluripotent stems cells, contain fewer gems (Coovert et al., 1997; Lefebvre et al., 1997; Ebert et al., 2009). The number of gems is inversely correlated with SMA severity (Coovert et al., 1997; Lefebvre et al., 1997; Patrizi et al., 1999). Furthermore, patient-specific fibroblasts treated with antisense oligonucleotides to correct aberrant *SMN2* splicing to increase SMN levels resulted in a significant increase in the number of gems (Al-Hilal et al., 2024).

In a mouse model the c.5C>G transgene causes mild SMA, and motor neurons from transgene mice are positive for nuclear gems, suggesting that the p.Ala2Gly SMN can form partially functional higher order SMN complexes (Monani et al., 2003). Analysis of SMN levels and nuclear gem quantities in tissues collected from human SMA patients with the c.5C>G variant have not yet been investigated.

Our aim was to investigate the clinical progression in two patients with the rare mild missense c.5C>G variant. Furthermore, we aimed to collect tissues from these patients and investigate levels of nuclear gems within these tissues.

## Materials and methods

### Ethics declaration

Results and available clinical information were acquired from patients included in this study; approval was granted by the Mayo Clinic Investigational Review Board (IRB # 19-004374) and informed consent from patients was obtained.

### SMA deletion/duplication analysis

The SMA deletion/duplication assay uses novel droplet digital PCR technology to accurately quantify *SMN1* exon 7 NC_000005.9 (NM_022874.2) and *SMN2* exon 7 NC_000005.9 (NM_022876.2) by counting copies of the c.744C in *SMN1* and the c.744T in *SMN2* (Vidal-Folch et al., 2018). QuantaSoft Analysis Pro version 1.0.596 (Bio-Rad) was used for droplet-cluster classification and Poisson function application was used for determination of absolute copy numbers (Vidal-Folch et al., 2018).

### *SMN1* full gene analysis

The *SMN1* full gene sequencing assay utilizes long-range PCR of exons 2–8 of *SMN1* gene NC_000005.9 (NM_000344.3) followed by bidirectional Sanger sequencing to identify variants. Exon 1 of *SMN1* gene NC_000005.9 (NM_000344.3) is PCR-amplified and bidirectionally Sanger-sequenced to identify variants in exon 1 (Vidal-Folch et al., 2018). Variants detected in *SMN1* exon 1 cannot be distinguished from variants in *SMN2* exon 1.

### Fibroblast isolation

Tissue was rinsed in collection media and placed in a 10 cm dish with media. The biopsy was minced and selected pieces were transferred to a dish and covered with a coverslip and covered in media. The plate was incubated for 4 days, and media was changed every 5–7 days. Once the coverslip became confluent, cultures were removed from the coverslip using trypsin and expanded in culture dishes.

### Tissue culture

Cultures were grown in DMEM media supplemented with 5% FBS. Media was changed every 1–2 days. Cultures were passaged using trypsin. Trypsin was inactivated by 1:1 dilution with media and centrifugation at 500 g for 4 min. Cultures were resuspended in media and plated.

### Immunofluorescence

Fibroblasts were fixed in 4.0% paraformaldehyde (Sigma Aldrich) solution in phosphate buffered saline (PBS), blocked and permeabilized using 0.1% Triton-X, 0.1% Tween-20 and 2.5% BSA in PBS. Primary antibody (BDBiosciences, at 1:100) to SMN was added, and counter stained with AlexaFluor secondary antibody (1:300). Nuclei were labelled with DAPI. Cells were imaged using Agilent BioTek Cytation.

### Quantification of nuclear gems

Nuclear gems per nuclei were identified and counted from two 40x immunofluorescence photomicrographs from two patients with the c.5C>G *SMN1* variant and an *SMN1* deletion, an SMA patient with homozygous *SMN1* deletion and *SMN2* copy numbers equal to the patients (two copies of *SMN2*), and a carrier control individual (one copy of *SMN1*). The carrier control individual is the mother of the SMA patient with homozygous deletion of *SMN1*.

## Results

### Clinical presentation

We identified two SMA patients with the rare missense pathogenic variant c.5C>G. We confirmed these patients have heterozygous deletion of exon 7 and heterozygous c.5C>G variant. These patients are siblings and they presented to the neurology clinic in adolescence with progressive muscle weakness. The older patient, a 16-year-old boy designated as Patient 1, suffered from a delay in gross motor milestones; only gaining the ability to roll over at 12 months of life, sit independently by 24 months of life, and walk by 30 months of life. He was notably slower than his peers as a child. He began suffering from frequent falls by 11 years of age and required a wheelchair around the time of the clinical evaluation. The younger patient, a 15-year-old girl designated as Patient 2, had normal gross motor milestones, but developed progressive weakness by 11 years of age. She was still ambulatory at the time of the clinical evaluation but required a wheelchair a few months later.

Both patients exhibited proximal muscle weakness that was more prominent in their lower extremities. Patient 1 had 4/5 strength in his biceps, triceps, and brachioradialis bilaterally, 4+/5 strength in his wrist extensors and digit extensors and flexors bilaterally. In his lower extremities, he had 2/5 strength in his iliopsoas, hip adductors, quadriceps, and hamstrings. His deep tendon reflexes were normal in his upper extremities. In his lower extremities, he had reflexes of 1+ throughout. He exhibited mild fine tremor in his hands bilaterally. Patient 2 had 4+/5 strength in her triceps and biceps bilaterally. In her lower extremities, she had 4+/5 strength of her iliopsoas and quadriceps. Her deep tendon reflexes were normal in the upper extremities. In her lower extremities, reflexes were 1+ at patella and ankle.

A muscle biopsy from Patient 2 and EMGs from both patients were performed at an external institution. The muscle biopsy diagnosis was reported as abnormal muscle with features of neurogenic atrophy. EMGs were consistent with a diffuse anterior horn cell disease.

Spinal muscular atrophy was suspected, and molecular testing was pursued. Results from deletion/duplication analysis showed that both patients had one copy of *SMN1*, consistent with SMA carrier status and not diagnostic of SMA. It was suspected they had a non-deletion pathogenic variant in their single copy of *SMN1*, and they were clinically diagnosed with SMA.

At age 36 and 34, they re-presented to the neurology clinic as new disease-modifying therapies had become available. At that time, both patients required a wheelchair and suffered from proximal weakness of the upper extremities. Neither patient was experiencing dysphagia, dyspnea, orthopnea, and neither required noninvasive ventilator support (Table 1).

**TABLE 1:** Comparison of c.5C>G Patients to SMA Patient with Matched *SMN2* Copy Number.

	Patient 1	Patient 2	Patient 3
SMA Type	3	3	1
*SMN1* CNV	1	1	0
*SMN1* Full Gene Analysis	c.5C>G	c.5C>G	N/A
*SMN2* CNV	**2**	**2**	**2**
Sex	Male	Female	Male
Current Age	39 years	38 years	6 years
Age at Onset	12 months	5 years	birth
Disease Progression	Delayed motor milestones: ambulation at 2.5 years	Normal motor milestones: ambulation at 1 year	Feeding difficulties at birth
	Frequent falls at 11 years	Could not climb stairs at 11 years	Muscle hypotonia at birth
	Loss of ambulation at 13 years	Loss of ambulation at 16 years	Respiratory distress requiring intubation at 3 months
Current Clinical Characteristics	No dysphagia	No dysphagia	Ventilator dependent
	No dyspnea or orthopnea	No dyspnea or orthopnea	G-tube dependent
	No noninvasive ventilator support at night	No noninvasive ventilator support at night	Can sit 2 min without support
	Can’t lift hands above head	Can lift hands above head	Good head control
	Progressive weakness of the hands	Progressive weakness of the hands	Can manipulate toys

### Molecular testing–*SMN1* full gene analysis

*SMN1* full gene analysis was pursued in an external diagnostic laboratory to search for the second variant. The patients were found to have one copy of *SMN1* and the missense variant c.5C>G in exon 1. However, the molecular testing could not distinguish *SMN1* exon 1 from *SMN2* exon 1. Both patients were found to have two copies of *SMN2*.

Of note, their clinical course is not consistent with the classic clinical course of SMA patients with two copies of *SMN2*. This difference in clinical phenotype is striking when compared to an SMA patient with the same copy number of *SMN2*, designated as Patient 3 (zero copies of *SMN1*, two copies of *SMN2*). This patient is a 6-year-old boy who presented at birth with feeding difficulties and hypotonia (Table 1). He exhibited respiratory distress requiring intubation at 3 months of age. Currently, he is ventilator and G-tube dependent (Table 1).

**FIGURE 1:**
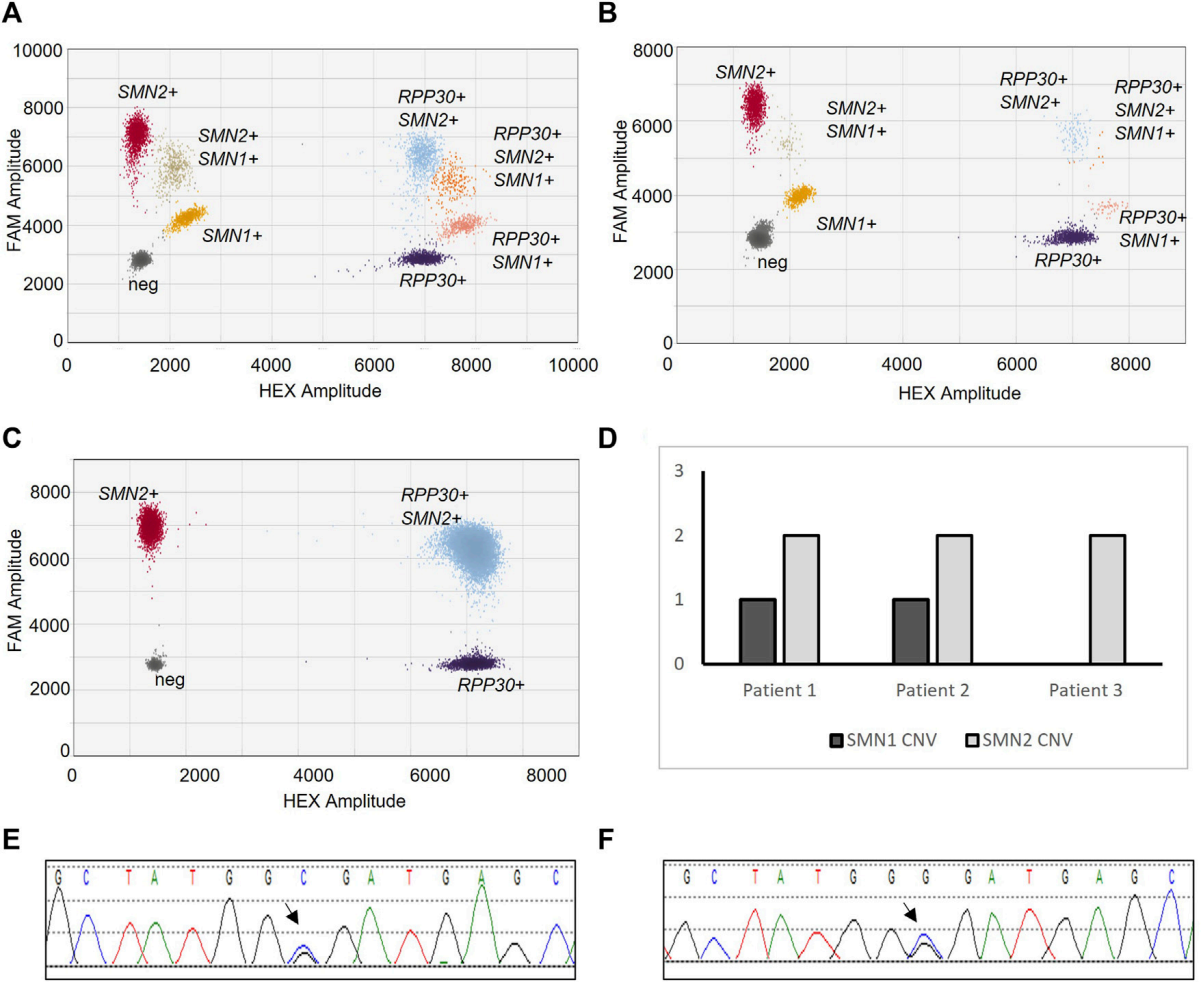
SMA Molecular Diagnostic Testing. SMA deletion/duplication analysis via ddPCR from Patient 1 **(A)**, Patient 2 **(B)**, and Patient 3 **(C)**. Droplet clusters correspond to populations of nucleotide targets; *SMN1*, *SMN2*, or *RPP30* (control). Resulting CNV of *SMN1* and *SMN2* for each sample **(D)**. Sanger sequencing electropherogram of *SMN1* from Patient 1 **(E)** and Patient 2 **(F)**. Arrow notes c.5C>G missense variant.

Diagnostic testing was repeated at our internal reference laboratory. Patient 3 was tested with Patient 1 and 2 as a control. ddPCR deletion/duplication analysis of Patients 1 and 2 revealed *SMN1* positive clusters, as compared to Patient 3 which did not have an *SMN1* positive cluster (Figures 1A–C). Absolute copy number analysis revealed that Patients 1 and 2 had one copy of *SMN1* and two copies of *SMN2* (Figure 1D) as compared to Patient 3 which had zero copies of *SMN1* and two copies of *SMN2* (Figure 1D).

*SMN1* full gene sequencing analysis was performed. Sanger sequencing detected the c.5C>G variant in Patients 1 and 2 (Figures 1E, F). The C allele was also detected. Importantly, the sequenced PCR reaction amplified both *SMN1* exon 1 and *SMN2* exon 1.

The mother of Patient 1 and 2 was also tested, and results from an external reference laboratory revealed two copies of *SMN1* and the missense variant c.5C>G in exon 1, also unable to distinguish *SMN1* exon 1 from *SMN2* exon 1.

Using this additional information, a family pedigree was constructed (Supplemental Figure S1). It is assumed that the patient’s father (not tested) is a traditional SMA carrier with an *SMN1* deletion, while their mother is likely an SMA carrier with two copies of *SMN1*, one copy with the missense variant c.5C>G. The affected patients inherited the allele with the deletion from their father and the allele with the missense variant from their mother (Supplemental Figure S1). Molecular testing and clinical information regarding the patients’ siblings were unavailable. Ultimately, the molecular tests could not render a definitive diagnosis. The patients were clinically diagnosed with SMA.

SMA patients with two copies of *SMN2* typically have severe disease (Type 1 SMA). However, patients with the c.5C>G missense variant and an *SMN1* deletion are known to have disease milder than their *SMN2* copy number would predict (Table 2). This variant is rare with scattered reports. All reported cases have one or two copies of *SMN2* (Table 2). All but three patients have mild disease and remain ambulatory into adolescence or even adulthood (Table 2).

**TABLE 2:** Reported Cases of *SMN1* c.5C>G.

Report	*SMN1* variant	SMA type	Number of patients	*SMN2* copy number	Phenotype
Parsons DW et al. Am. J. Hum. Genet. 1998	c.5C>G	Type III	2	Not reported	Not reported
		Type II	1	Not reported	Not reported
Yamamoto T et al. Brain Dev. 2014	c.5C>T	Type III	2	1 *SMN2* CNV	Ambulatory through adolescence and adulthood
Bai JL et al. Genet Test Mol Biomarkers. 2014	c.5C>G	Type III	1	2 *SMN2* CNV	Not reported
Kubo Y et al. J Hum Genet. 2015	c.5C>T	Type III	1	1 *SMN2* CNV	Ambulatory through adolescence and adulthood
Mendonça RH et al. Neurol Genet. 2020	c.5C>G	Type II	3	1 *SMN2* CNV	Non-sitter, requires wheelchair assistance
		Type III	6	1 *SMN2* CNV	Ambulatory through late childhood, adolescence, and adulthood

### Patient-specific analysis of nuclear gems

SMN immunofluorescence staining of skin fibroblasts was performed on Patients 1-3, along with a healthy SMA carrier control (one copy of *SMN1*). The SMA carrier control is the mother of Patient 3.

**FIGURE 2:**
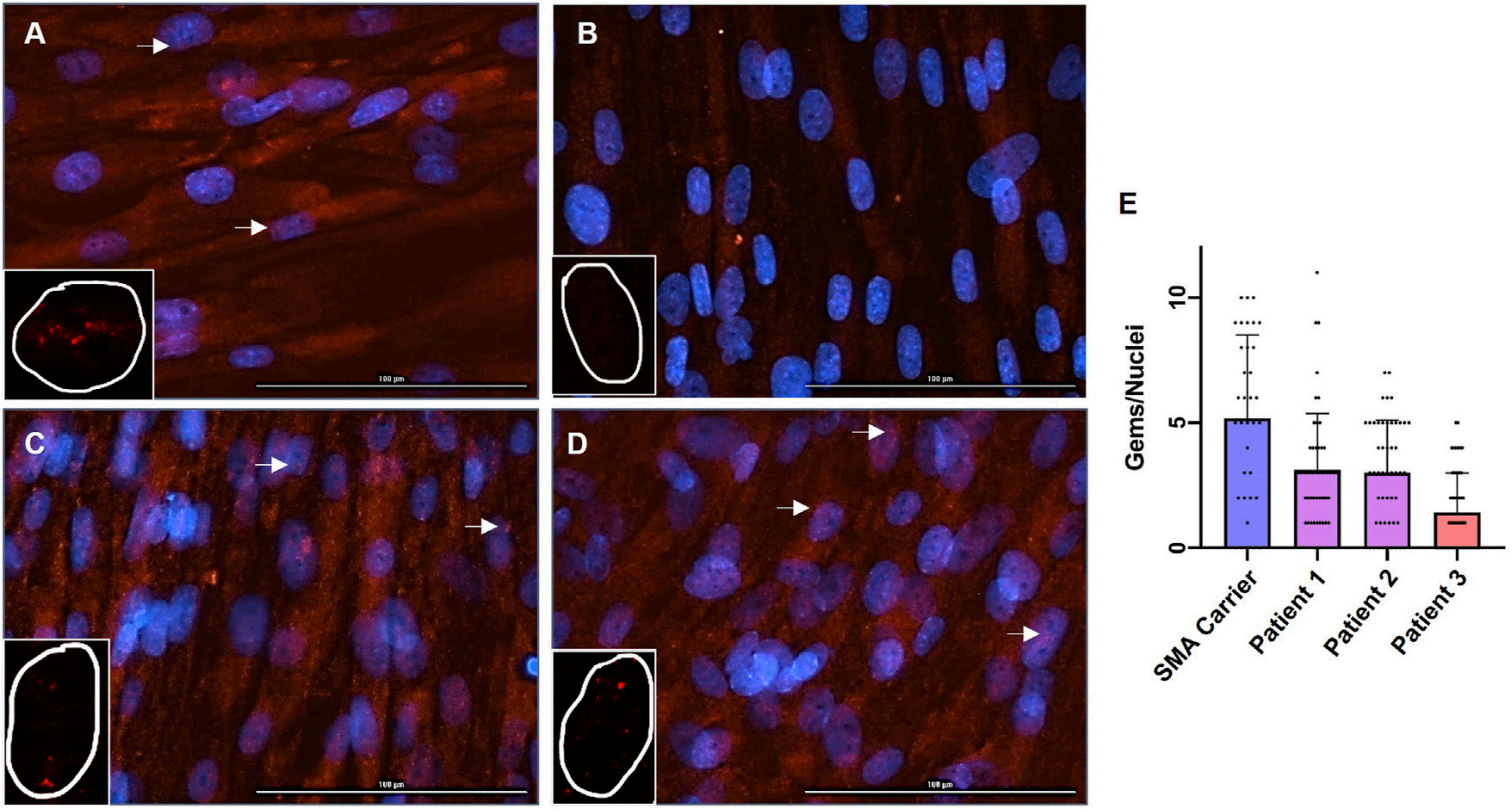
Immunofluorescence Staining of SMN in Fibroblasts and Quantification of Nuclear Gems. Immunofluorescence staining for SMN (red) and DAPI (blue) in patient-specific fibroblasts; **(A)** SMA Carrier, **(B)** Patient 3, **(C)** Patient 1, and **(D)** Patient 2. The arrow denotes gems. ×20 magnification. Bar = 100 uM. Inset with representative SMN staining (red) of single nuclei. Identified gems were quantified and normalized to total nuclei **(E)**; SMA Carrier: mean = 5.17, n = 35, standard error from the mean (SEM) = ± 0.56, Patient 1: n = 52, mean = 3.11, SEM = ± 0.30, Patient 2: mean = 3.00, n = 50, SEM = ± 0.21, Patient 3: mean = 1.41, n = 58, SEM = ± 0.31. A one-way ANOVA was performed to reveal there was a statistically significant difference in mean gems per nuclei between the fibroblast cultures [F(3, 191) = (19.84), *p*-value < 0.00001].

SMN levels in fibroblasts from Patient 1 and Patient 2 are higher than SMN levels in fibroblasts from Patient 3 (Figures 2A–D). Furthermore, like the fibroblasts from the SMA carrier control, the fibroblasts from Patient 1 and Patient 2 are positive for multiple nuclear gems per nuclei (Figures 2A–D). Quantification of gems revealed that nuclei of fibroblasts from Patient 1 contained a mean of 3.1 gems per nuclei, and fibroblasts from Patient 2 contained a mean of 3.0 gems per nuclei. This is compared to a mean of 5.2 gems per nuclei in the SMA carrier control fibroblasts and 1.4 gems per nuclei in Patient 3 fibroblasts (Figure 2E).

A one-way ANOVA revealed that there was a statistically significant difference in mean gems per nuclei between the fibroblast cultures [F(3, 191) = (19.84), *p*-value < 0.00001]. Tukey’s HSD Test for comparisons found that the mean gems per nuclei was significantly different between the SMA carrier control and Patient 3 (*p* < 0.00001). The mean gems per nuclei was significantly different between the SMA carrier control and Patient 1 and Patient 2; Patient 1 and Patient 2 (*p* = 0.00012 and *p* = 0.00004 respectively). The mean gems per nuclei was significantly different between Patient 3 and Patient 1 and Patient 2; Patient 1 and Patient 2 (*p* = 0.00220 and *p* = 0.00509 respectively). There was no statistically significant difference in mean gems per nuclei between Patient 1 and Patient 2 (*p* = 0.99483).

## Discussion

The patients described here have the heterozygous c.5C>G variant, a heterozygous deletion of exon 7, and two copies of *SMN2*, however, they exhibit a mild clinical course that is not consistent with patients with two copies of *SMN2*. This is highlighted by direct comparison to an SMA patient with homozygous deletion of exon 7 and two copies of *SMN2* and a typical clinical course (Table 2). Of note, this patient is not genetically related to the patients with the c.5C>G variant and their different genetic backgrounds may contribute to some variability in phenotype.

Interestingly, the clinical presentation of the two patients is mildly discordant. The female patient presented with a later age of onset and remained ambulatory longer than the male patient. Sex-specific phenotypic variability has been reported in SMA (Singh et al., 2021; Zhao et al., 2021). Non-clinical and clinical reports have shown that SMA may affect the male reproductive system (Ottesen et al., 2016; Howell et al., 2017; Lipnick et al., 2019; Bar-Chama et al., 2024). Furthermore, many known disease modifiers are encoded by genes on the X chromosome, and overexpression of modifiers results in milder phenotypes in female patients (Oprea et al., 2008; Han et al., 2012; Powis et al., 2016).

The missense variant c.5C>G occurs in exon 1 of *SMN1*. Exon 1 of *SMN1* is identical to exon 1 of *SMN2*, making the design of full gene analysis challenging. Most clinical tests utilizing long range PCR only amplify exons 2a-7. The patients investigated here did not receive a definitive diagnosis from an outside reference laboratory, and our internal diagnostic testing has the same limitation. Most clinical diagnostic tests cannot definitively molecularly diagnose exon 1 variants that cause SMA. This is a significant limitation in SMA diagnostic testing.

The mutation spectrum of *SMN1* includes pathogenic alterations in exon 1 of *SMN1*, including missense variants, duplications, insertions, and deletions (Fokkema et al., 2011; Qu et al., 2016). In a review of cases referred to our laboratory in the last six years, we have detected nine *SMN1* variants, three of which occurred in exon 1; a known pathogenic frameshift variant, a novel pathogenic deletion, and missense variant classified as a variant of unknown significance (data not shown). Now, with new disease-modifying therapies available to patients, it is critical to accurately detect all pathogenic alterations in *SMN1*.

Methods utilizing long-range PCR that can amplify exons 1–8 of *SMN1* have been developed (Kubo et al., 2015). In fact, this method successfully detected the c.5 > G variant in exon 1 of *SMN1* (Kubo et al., 2015). Furthermore, some clinical assays have been optimized to use long range PCR to differentiate the full coding region of *SMN1* from *SMN2* (Miller et al., 2023). This method effectively detected the c.5C>G variant in exon 1 of *SMN1* (Miller et al., 2023). There is a need for molecular diagnostic labs to optimize current diagnostic methods to differentiate *SMN1* exons 1–8 from *SMN2* exons 1–8.

Long-read sequencing is a potential future method for clinical diagnostic testing. Long-read PacBio HiFi sequencing and subsequent analysis with Paraphase, an informatics method that identifies *SMN1* and *SMN2* haplotypes, detects pathogenic variants, determines the copy number variation, and detects phased variants (Chen et al., 2023). Another HiFi long read sequencing approach effectively identified *SMN1* and *SMN2* single nucleotide variants, small insertions and deletions, and large *SMN1* deletions. This method achieved 100% accuracy for *SMN1* copy number and 99.5% accuracy for *SMN2* copy number (Bai et al., 2023). Long read technology will ultimately lead to more comprehensive SMA molecular diagnostic testing.

Both optimized long-range PCR based sequencing and long read sequencing technology detects the *SMN1* c.5C>G variant. This variant is significant as it is known to cause less severe disease than *SMN2* copy number would predict (Table 2). Our data reinforces this discordant phenotype (Table 1). Furthermore, mice with the c.5C>G (p.Ala2Gly) transgene have a mild SMA (Monani et al., 2003). A mild phenotype indicates that p.Ala2Gly SMN is partially functional, thereby preventing the development of a severe phenotype.

Nuclei of motor neurons collected from p.Ala2Gly transgenic mice contain nuclear SMN gems, indicating that p.Ala2Gly SMN can form nuclear complexes. To the best of our knowledge, levels of SMN and nuclear gems in tissues collected from patients with the c.5C>G variant have not yet been previously reported. Our novel data from human tissues are in keeping with the data obtained from p.Ala2Gly transgenic mice. Our data revealed that SMN levels in fibroblasts from Patient 1 and Patient 2 are higher than the SMN levels in fibroblasts from Patient 3, and are comparable to SMN levels in fibroblasts from a carrier control. This data is consistent with a prior study showing that *SMN1* transcript levels isolated from a patient with the c.5C>G variant were not reduced compared to a healthy carrier control (Bai et al., 2014). Furthermore, nuclei of fibroblasts from Patient 1 and Patient 2 contained nuclear gems. The gem number is higher than that observed in Patient 3, but fewer than that observed in the carrier control. Of note, *SMN2* copy number of the carrier control is unknown. In addition, Patient 3 and the healthy carrier control are not genetically related to Patient 1 and Patient 2, and their different genetic backgrounds may contribute to differences in SMN levels and numbers of nuclear gems. Even so, this suggests that the p.Ala2Gly SMN cannot form the same quantity of gems as wild-type SMN. This is consistent with prior reports that the quantity of gems correlates to disease severity (Coovert et al., 1997; Lefebvre et al., 1997; Patrizi et al., 1999). Overall, such findings indicates that the p.Ala2Gly affects SMN interactions and ultimately fully functional gem formation.

Exon 1 of *SMN1* codes for the N-terminus of SMN and is just proximal to the Gemin-2 binding domain which is important for SMN complex assembly (Lorson and Androphy, 1998; Otter et al., 2007; Ogawa et al., 2009; Zhang et al., 2011). However, the utmost N-terminal is not known to be responsible for vital interactions needed for oligomerization or higher order complex formation. Experiments performed using an immortalized mouse embryonic fibroblast (iMEF) line in which Smn can be conditionally deleted using Cre recombinase revealed that the p.Ala2Gly SMN did not rescue survival in the conditionally deleted iMEFs, suggesting the SMN function that is lost with this variant is essential for cell function and survival (Blatnik et al., 2020). When the p.Ala2Gly SMN was co-expressed with many other missense variants, iMEF survival and snRNP assembly was rescued. The only variant tested that did not rescue survival is the p.Thr274Ile (Blatnik et al., 2020). p.Thr274Ile is coded by exon 6 and is in the YG-box oligomerization domain (Lorson et al., 1998). Like p.Ala2Gly, p.Thr274Ile is also a missense variant that causes disease milder than *SMN2* copy number would predict (Hahnen et al., 1997). Collectively, this suggests a role that is shared by the N-terminus and C-terminus of SMN. This shared interaction and function does not preclude partially functional complex formation but does limit SMN function by limiting fully functional complex formation to wild-type levels.

In addition to SMN’s role in RNA metabolism, SMN has been known to be involved in multiple cellular functions, including macromolecular trafficking, stress granule formation, cell signaling, and cytoskeleton maintenance, and not all of these functions require the canonical SMN complex (Singh et al., 2017). The N-terminus may have an important role in multiple SMN-dependent cellular functions and may have important SMN complex-independent interactions. As such, the c.5C>G variant may alter these functions and interactions.

It is also important to consider the effects of the c.5C>G variant on the transcription of *SMN1*. Exon 1 variants proximal to the promoter may affect transcription initiation and transcript stability, and possibly translation and protein stability. Future studies include measuring *SMN1* transcript levels and SMN protein levels isolated from the patient’s fibroblasts. Recently, an *SMN2* super minigene was generated to investigate the regulation of *SMN2* transcription (Ottesen et al., 2024). This super minigene was utilized to recapitulate the splicing pattern of endogenous *SMN1* with a pathogenic variant (Ottesen et al., 2024). Using such a construct may address questions regarding the effects of the c.5C>G variant on transcription.

Taken together, this data suggests there are discrete functions of SMN that can regulate motor neuron survival and function and investigating the p.Ala2Gly SMN will lead to a greater understanding of SMN function. Investigating the c.5C>G variant, including protein and transcript levels resulting from the c.5C > G variant, differential components of p.Ala2Gly SMN complex, and differential gene expression resulting from p.Ala2Gly SMN complexes, will reveal novel SMN complex interacting partners with discrete functions crucial to SMN function and subsequent altered gene expression that is critical for motor neuron function and survival.

## Data Availability

The datasets presented in this article are not readily available because of ethical and privacy restrictions. Requests to access the datasets should be directed to the corresponding author.

## Supplementary material

Supplementary Figure S1 Family Pedigree of Patients. Arrows designate SMA patients. I2, father of the proband, indicates SMA carrier status, heterozygous deletion of *SMN1*, and I3, mother of proband indicates SMA carrier status, heterozygous c.5C>G pathogenic missense variant in exon 1.
