# Supplementary figures and images for: SMN1 c.5C>G (p.Ala2Gly) missense variant, a challenging molecular SMA diagnosis associated with mild disease, preserves SMN nuclear gems in patient-specific fibroblasts
Source: Front Genet. 2024 Jul 30;15:1406819. doi: 10.3389/fgene.2024.1406819 (PMC11319185; doi:10.3389/fgene.2024.1406819)

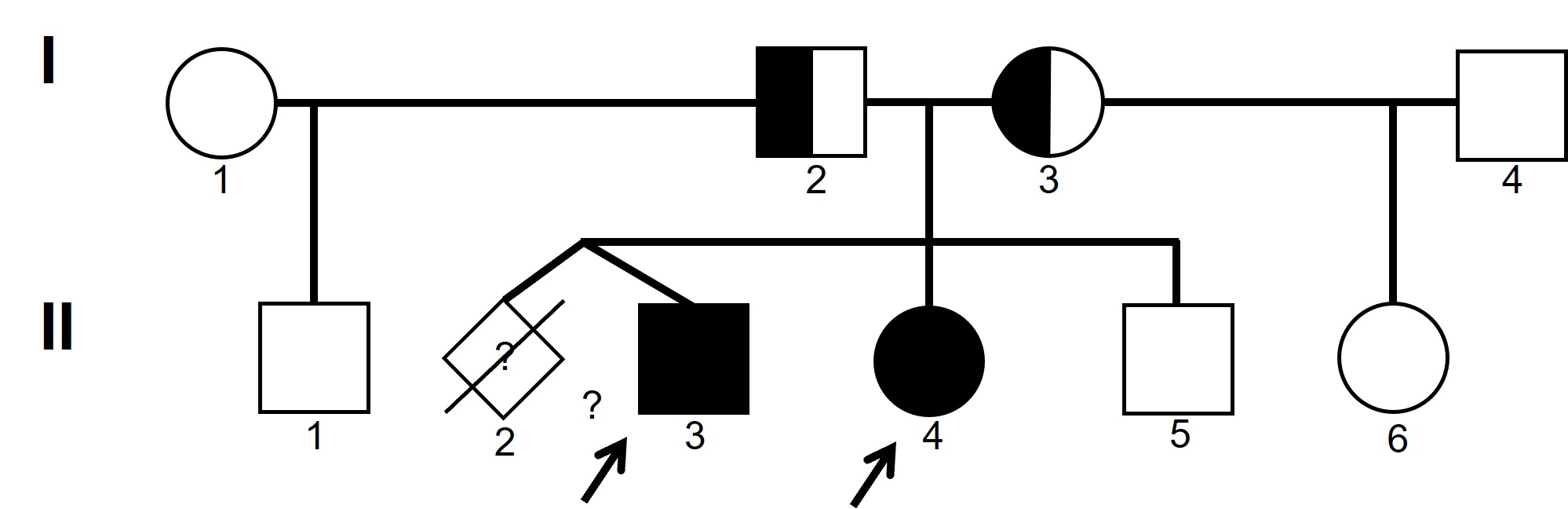

Supplement: Supplementary file 1 [file Image1.jpg]
